# Process Optimization of Phytoantioxidant and Photoprotective Compounds from Carob Pods (*Ceratonia siliqua* L.) Using Ultrasonic Assisted Extraction Method

**DOI:** 10.3390/molecules27248802

**Published:** 2022-12-12

**Authors:** Radia Ayad, Rima Ayad, Hayat Bourekoua, Mostefa Lefahal, El Hani Makhloufi, Salah Akkal, Kamel Medjroubi, Gema Nieto

**Affiliations:** 1 Valorization of Natural Resources, Bioactive Molecules and Biological Analysis Unit, Department of Chemistry, Frères Mentouri Constantine 1 University, Constantine 25000, Algeria; 2 Laboratory of Phytochemistry and Pharmacology, Department of Chemistry, Faculty of Exact Sciences and Informatics, University Mohammed Seddik Benyahia of Jijel, Jijel 18000, Algeria; 3 Laboratory of Biotechnology, Environment and Health, Department of Applied Microbiology and Food Science, Faculty of Nature and Life Sciences, University Mohammed Seddik Benyahia of Jijel, Jijel 18000, Algeria; 4 Laboratoire de Nutrition et Technologie Alimentaire (LNTA), Institut de la Nutrition, de l’Alimentation et des Technologies Agro-Alimentaires (INATAA), Frères Mentouri-Constantine 1 University, Constantine 25000, Algeria; 5 Department of Food Technology and Human Nutrition, Faculty of Veterinary Sciences, Regional Campus of International Excellence “Campus Mare Nostrum”, University of Murcia, 30071 Murcia, Spain

**Keywords:** ultrasonic, Carob byproducts, optimization, traditional methods, antioxidant, photoprotective

## Abstract

The current study first describes the extraction of phytoantioxidant polyphenols from Carob byproducts (pods) using maceration and heating-assisted extraction as traditional methods and ultrasonic-assisted extraction (UAE) as an innovative method to determine the most efficient extraction process in terms of four targeted responses: total phenolic content (TPC), antioxidant activities (TAC and DPPH), and photoprotective properties as measured by the sun protection factor (SPF). Second, we used response surface methodology (RSM) with a central composite rotatable design (CCDR) approach to investigate the influence of process variables (extraction time, extraction temperature, and solvent concentration) on UAE, which was found to be the most effective extraction technique in our study. Carob byproduct extracts had a TPC ranging from 6.21 to 21.92 mg GAE/g dw, a TAC ranging from 22.00 to 49.30 mg AAE/g dw, DPPH scavenging activity ranging from 56.35 to 90.50%, and SPF values ranging from 8.62 to 22.37. The optimal UAE conditions for maximum TPC, TAC, DPPH, and SPF responses were determined to be 38.90% ethanol, 53.90 °C, and 50.92 min. Using Carob as a source for sustainable and bioactive products in conjunction with optimized UAE is a promising contribution to the cosmetic industry that will help to strengthen the concept of environmentally-friendly “green chemistry”. Given that Carob pulp or seeds are considered food byproducts, the research presented here encourages the use of these agri-food waste materials in cosmetics.

## 1. Introduction

As the body’s largest organ and also known as the body’s armor, the skin provides a strong epithelial barrier that protects internal organs from the harmful effects of environmental exposures such as chemical oxidants, mechanical damage, and solar ultraviolet radiation (UVR) [1]. UVR (290–400 nm) reaching the earth’s surface acts as a mediator to activate several signaling cascades in skin, resulting in the generation of reactive oxygen species (ROS) [2,3]. UV-generated ROS can oxidatively destroy or deplete the skin’s antioxidant defense system, resulting in a variety of dermal disorders such as sunburns, pigmentation changes, wrinkles, premature aging, and skin cancer. Therefore it is now more important than ever to provide our skin with an effective photoprotection [3,4,5]. Broad-spectrum sunscreens with the appropriate sun protection factor SPF are now the mainstay of many studies for reducing UV’s detrimental effects. However, the photostability, toxicity, and damage to marine ecosystems of most artificial sunscreen constituents limit their efficacy and safety [6,7,8]. In recent years, the emphasis has shifted to other protective measures with new strategies. Botanical antioxidant extracts and DNA repair enzymes applied topically are examples of these [9,10,11,12]. It has been suggested that incorporating antioxidants into sunscreens can improve their photoprotective qualities and offer greater protection [4,13,14].

Fruits, vegetables, food byproducts, and natural plant extracts containing phytoconstituents, particularly polyphenols, have long been used to treat a wide range of skin conditions. Due to their antioxidant properties, botanical phenolic extracts are effective in treating skin disorders, as free radical species are one of the leading causes of skin damage [15,16,17,18,19]. Polyphenols are a type of compound found in many plants that has anti-inflammatory, immunomodulatory, anti-oxidative, and sun protection properties [15,20].

Polyphenols, in fact, have a structural similarity to synthetic UV filters, and they hold great promise for widespread development and application in sunscreens as new photoprotective and antioxidant agents [15,20,21,22]. In parallel, the industrial sector including cosmetic, pharmaceutical, food and agriculture is heavily involved in the search for green and natural sources of valuable compounds such as polyphenols for use as potential alternatives to toxic synthetic chemicals in order to meet the evolving consumer demand for products beneficial to health as well as global environmental concerns. Aloe Vera extract, for instance, has proven its efficacy in the food, pharmaceutical, and cosmetic industries, as well as being investigated as a good alternative to chemical pesticides in agriculture [23].Nowadays, sustainability, reducing environmental impact, and making efficient use of energy resources are all becoming increasingly important, and the value of environmental and human-friendly botanical ingredients is expected to rise [24,25,26,27].

Thus, there is a major focus on overcoming the challenge to render more efficient the extraction of these natural extracts from their complex plant matrices. Conventional methods such as maceration, heating-assisted extraction, Soxhlet extraction, distillation, and infusions have been used for many years. Typically, they are not eco-friendly due to the large amounts of solvents and energy required for their process which, as well as a lack of sustainability and green extraction protocols, also raises concerns about worker and consumer safety [28,29]. With a commitment to developing green and clean technologies, new extraction processes for recovering valuable compounds are emerging. Ultrasound-assisted solvent extraction (UAE) is one of the most recent methods for extracting bioactive constituents such as polyphenols from raw materials such as agri-food byproducts. Compared to traditional extraction methods, the ultrasound-assisted extraction method offers several advantages including shorter extraction time, less amount of solvent and higher extraction yields [30,31].Ultrasounds exert a mechanical effect; using cavitation generated by compression and expansion of ultrasonic waves, this phenomenon improves the penetration of the solvent into the sample matrix and increases the contact surface between the solid and liquid phases, which leads to higher diffusion and mass transfer. Using ultrasound, full extraction can be completed in a few minutes with high reproducibility, reduced solvent consumption, and simplified work-up [32]. Many investigations have proved that ultrasound-assisted extraction was successfully applied for liquid extractions of nutraceuticals [33], polysaccharides and polyphenols from their matrices [34,35,36]. As an industrial success story, Arkopharma laboratories have studied and developed a new process for the extraction of medicinal plants using ultrasonic cavitation. Their research found that ultrasound has a detexturation effect on the plant matrix, allowing for increased extraction and mass transfer. The results showed that yield concentration increased by 73%, while energetic consumption and environmental impact decreased by 25% and 33%, respectively [32,37].

Carob (*Ceratonia siliqua* L.), a well-known Mediterranean legume, is traditionally used for the production of animal feed. Nowadays, numerous reports support its use in food, pharmaceutical, and cosmetic industries [38]. The carob pod is composed of seeds (10–20%) which are considered the valuable part due to the extraction of locust bean gum; and the pulp (80–90%), considered a byproduct of the fruit processing or food industry. By contrast, in the pharmaceutical field, products obtained from de-seeded pods possess interesting therapeutic functions, while the seeds themselves remain a “food byproduct” [38,39,40,41].

In recent years, as a source of sustainable ingredients and products, carob has received extensive attention in scientific research because of its high nutritional value and potential beneficial health outcomes such as antibacterial, analgesic, lipid-lowering, anti-cardiovascular, proapoptotic, anti-cancer, anti-proliferative, cytotoxic, and antioxidant properties [39,40,41,42,43,44,45,46,47].Both the carob pulp and seeds were discovered to be high in phenolic compounds with high antioxidant potential and ability to scavenge radicals [38]. Gallic acid, (+)—catechin, (−)—epicatechin, (−)—epicatechin gallate, (−)—epigallocatechin, (−)—epigallocatechigallate, myricetin, quercetin and their derivatives, and tannin compounds are the main polyphenolic compounds found in carob parts [43].

Taking the aforementioned evidence and research findings into consideration, the goal of our study is to compare the effectiveness of the ultrasound assisted extraction process on the recovery of total phenolic content, antioxidant activities, and photoprotective potential by measuring SPF of carob pods extract (pulp and seeds) with conventional techniques of maceration and heating-assisted extraction. Thereafter, using a surface response methodology (RSM) and a central composite rotatable design (CCDR), we attempted to optimize ultrasound-assisted extraction (UAE) of the prepared extract by investigating the influence of process parameters (extraction time, extraction temperature, and solvent concentration). Despite the fact that the polyphenolic profile of carob parts and their activity have been extensively reported, there has been no discussion of the photoprotective activity of carob pod extract, nor has there been a detailed study of the effect of optimizing the ultrasound processing parameters on its SPF values, and that is what gives originality to our work. As sustainability is involved in our study, the circular bio-economic process and zero waste are central to our research as well. Given that either carob pulp or seeds are considered food waste, the study presented herein can contribute to alternative approaches for recycling food waste into valuable products in cosmetics; consequently, it can promote a circular bio-economy by adding new utilization of carob byproducts over the long term with a conservation of product value, materials, and resources in the economy with minimal waste generation [48].

## 2. Results and Discussion

### 2.1. Selection of Efficient Extraction Process

**Figure 1:**
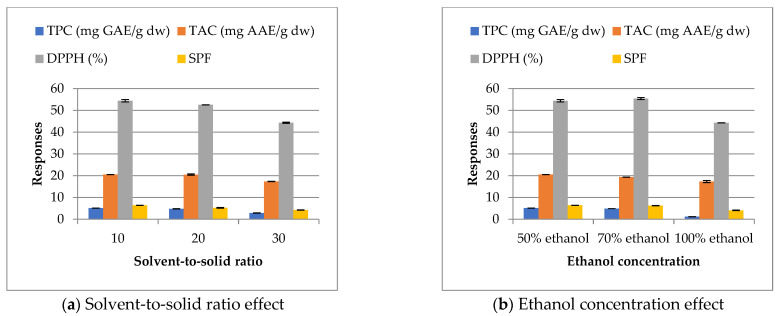
Effect of solvent-to-solid ratio and ethanol concentration on responses (maceration at 25 °C for 60 min).

At the outset of our study, we performed preliminary assays for selecting the most efficient extraction method of carob byproducts. Although there is no significant disparity in the obtained responses (TPC, TAC, DPPH, and SPF) except for those of 100% ethanol (Figure 1b), the solvent-to-solid ratio (10/1; *v*/*m*) and 50% ethanol concentration are considered the best to be used in the subsequent experiment (Figure 1). The greatest efficiency achieved with the maceration method was for 60 min: under those conditions, the amount obtained was 5.10 ± 0.02 mg GAE/g dw of TPC, a level of 20.54 ± 0.03 mg AAE/g dw of TAC, an inhibition of 54.40 ± 0.56% for DPPH, and a value of 6.42 ± 0.02 for SPF. Ethanol–water mixtures were found to be more effective for the extraction of antioxidants from botanical materials. Apart from being a non-toxic food-grade organic solvent, ethanol is also suitable for use in cosmetics [49].

**Figure 2:**
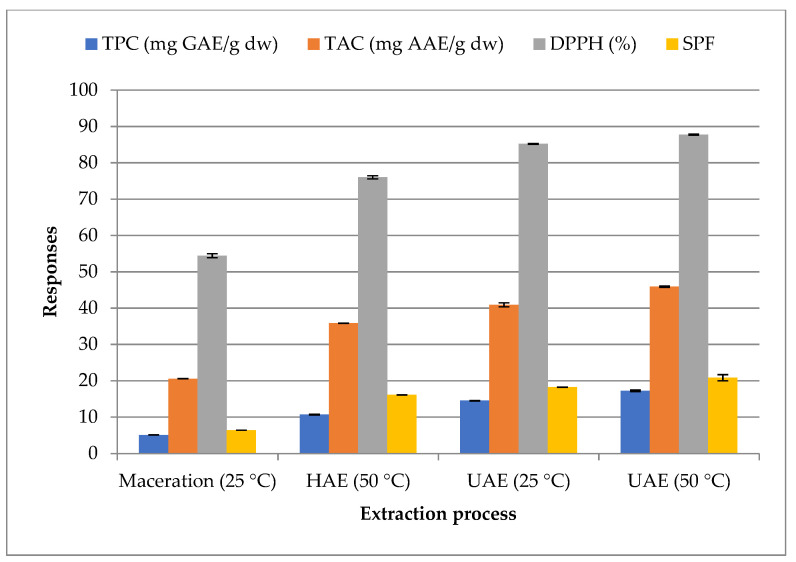
Effect of extraction process and temperature on responses. HAE—heating-assisted extraction, and UAE—ultrasound-assisted extraction.

To achieve the purpose of this work, the efficacy of ultrasound-assisted extraction (UAE) as a green method was compared with that of tow conventional methods: maceration and heating-assisted extraction (HAE). According to the results displayed in Figure 2, the effectiveness of the extraction methods for all variables was ranked by the measured responses (TPC, TAC, DPPH and SPF) in the following order: UAE > HAE > maceration.

In both conventional and ultrasonic methods, increasing the extraction temperature results in a marked increase in the responses of Carob byproduct extracts. The HAE responses were found to be higher with a quantity of 10.68 ± 0.13 mg GAE/g dw of TPC, a level of 35.86 ± 0.03 AAE mg/g dw of TAC, an inhibition of 76.03 ± 0.43% for DPPH, and a value of 16.09 ± 0.013 for SPF compared to those of maceration responses; but they remained lower than those recorded in UAE (at 25 °C and 50 °C). It is important to highlight that ultrasound enhanced the extraction yield compared to conventional methods. This latest outcome was demonstrated by the increase in all tested responses under UAE at 50 °C compared to those recorded under heating-assisted extraction for the same extraction time and temperature. UAE responses under 50 °C reached maximum levels for 17.23 ± 0.20 mg GAE/g dw of TPC, 45.9 ± 0.19 AAE mg/g dw of TAC, 87.74 ± 0.14% for DPPH scavenging assay, and a value of 20.82 ± 0.86 for SPF, within this screening. Our results are corroborated by those of Jovanović et al. [50].This work suggests that UAE could be the more valuable extraction process for obtaining photoprotective and antioxidant compounds from carob byproducts. After this preliminary study, we decided to optimize this technique, to better contribute to the promotion of the valorization of antioxidants in Carob byproducts as an important and safer alternative to chemical ingredients in cosmetics.

The effect of selected parameters (extraction time, extraction temperature, and solvent concentration) on the UAE extraction process of Carob byproducts was investigated in order to maximize their photoprotective potential by measuring SPF of the obtained solution while highlighting total phenolic content and antioxidant activities.

To the best of our knowledge, this is the first paper describing both the photoprotective potential of Carob byproduct extracts, and the influence of investigated process parameters on this activity in terms of SPF measurements using UAE and surface response methodology (RSM).

Within the experimental design, the measurement of each response (total phenolic content, antioxidant activities, and photoprotective properties) was carried out. Table 1 shows the decoded values and results of 20 experiments. Carob powder extracts had a total phenolic content (TPC) ranging from 6.21 to 21.92 mg GAE/g dw, a total antioxidant capacity (TAC) ranging from 22.00 to 49.30 mg AAE/g dw, DPPH scavenging activity ranging from 56.35 to 90.50%, and a photoprotective activity (SPF) ranging from 8.62 to 22.37.

### 2.2. Effect of Process Variables on Total Phenolic Compounds (TPC)

Table 1 shows the total phenolic content (TPC) of Carob powder extracts obtained through UAE. Regression analysis was performed on the experimental data, and model coefficients were evaluated for significance in phenolic compound extraction. This method’s coefficient of determination (*R*^2^) was 98.08%, indicating an adequate correlation between the model and the experimental results for the chosen parameters (Table 2). For the extraction of phenolic compounds, the following equation represents the relationship between extraction time, extraction temperature, and ethanol concentration:

(1) $$\begin{array}{c} \mathrm{TPC}\;=-55.76+0.441X_{1}+2.239X_{2}+0.254X_{3}-0.004908X_{1}{}^{2}-0.01467X_{2}{}^{2}+0.001471X_{3}{}^{2}\\ \;\;\;\;\;\;\;\;\;\;\;\;\;\;\;\;\;\;\;\;\;\;\;\;\;\;\;\;\;+0.00271X_{1}X_{2}-0.001146X_{1}X_{3}-0.01209X_{2}X_{3} \end{array}$$

**Figure 3:**
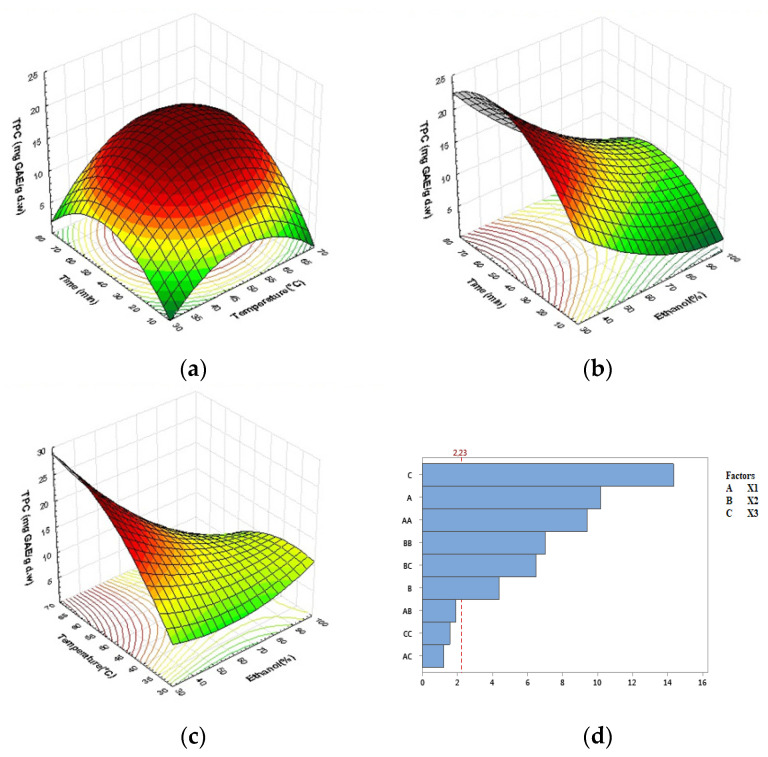
Response surface plots indicating combined effects of UAE variables on TPC: (**a**) time and temperature, (**b**) time and ethanol concentration, (**c**) temperature and ethanol concentration, (**d**) Pareto chart (α = 0.05).

According to the coefficients of the above equation and *p*-values in Table 2, all parameters (linear, quadratic, and interaction) had a significant effect on phenolic compound extraction (*p* < 0.0001). Furthermore, ethanol concentration has the most positive influence on TPC, followed by extraction time, squared term of extraction time, squared term of extraction temperature, interaction of ethanol concentration and extraction temperature, and finally extraction temperature, according to Pareto results (Figure 3d). Extraction conditions in quadratic and interaction terms had both negative and positive effects. Negative quadratic terms of extraction time and extraction temperature indicated that the saddle point of TPC began to decrease, whereas ethanol concentration revealed positive quadratic effects on TPC extraction.

Surface plot analysis in Figure 3 is consistent with multiple regression analysis. TPC content increases with extraction time and temperature, as shown in Figure 3a; a higher phenolic content was obtained at extraction temperatures ranging from 55 to 60 °C and extraction times ranging from 50 to 60 min. Following that, it began to fall.

In general, higher temperatures in solid–liquid extraction improve extraction efficiency and lead to higher recoveries of phenolic compounds by increasing their solubility, increasing diffusion rates, improving mass transfer and extraction yield, and reducing solvent viscosity and surface tension. However, prolonged exposure to high temperatures may result in lower levels of phenolics, indicating possible degradation and decomposition of these compounds [51,52,53]. Figure 3b depicts the effects of extraction time and ethanol concentration on TPC at a fixed temperature of 50 °C. In addition to its positive linear effect, extraction time is a more significant quadratic variable than others. According to this analysis, TPC levels reached an optimum between 35% and 60% ethanol in the range of 40–70 min. The lowest concentration of phenolics was found above 70 min, with a higher ethanol concentration (>65%).

For the effects of temperature and ethanol concentration (Figure 3c), concentrations of 30% to 50% ethanol combined with higher temperatures (50–60 °C) produced the poorest levels of TPC. The highest TPC value (TPC = 21.92 mg GAE/g dw) was obtained under the following experimental conditions: 50% ethanol concentration, 60 min of extraction time, and a temperature of 60 °C. The increase in extractable polyphenol quantities with the addition of water to ethanol can be explained by an increased swelling of plant materials and greater surface area between the plant matrix and the solvent, which improves mass transfer by diffusion [33,53,54]. Although there is a link between solvent extraction and maximum polyphenol extraction yield, it is clear that health and environmental risks must be considered in the food, pharmaceutical, and cosmetic industries. As a result, it may be advantageous to select an alternative and sustainable extraction solvent. In our experiment, we used ethanol and water, as well as UAE, which was consistent with green extraction. Ethanol is less harmful to the environment than other organic solvents, and only a small amount is required for UAE [49,54,55,56].

Data on the extraction of Carob’s phenolics is scarce in the literature, making direct comparison difficult due to differences in extraction methods and conditions such as solvent, process, and part of the material studied (extract or residue). Additionally, other studies show that the phenolic profiles of Carob vary depending on geographical origin, variety, cultivation, degree of maturation and conditions of extraction. The maximum levels of TPC in our study were closer to those previously reported by Avallone et al. [57] whose reported TPC ranged from 15.80 to 24.40 mg GAE/g; Roseiro et al. [58]—20.40 mg GAE/g by using UAE and 27.10 mg GAE/g by using supercritical fluid extraction; and Mansouri et al. [59]—20.38 mg GAE/g by using UAE; as well as Amrani et al. [60], who recently reported the TPC values of 14.713 mg GAE/g and 19.25 mg GAE/g in Carob seed extract using cold maceration and Soxhlet extraction, respectively [57,58,59,60].

A few studies on the optimized extraction of Carob polyphenols have been published. Almanasrah et al. [61] devised a two-step water extraction method to achieve an optimum value of 19.00 mg GAE/g Carob dry matter [61]. This result is lower than that reported by Roseiro et al. [62] for optimized decoction (39.50 mg GAE/g Carob dry matter) and optimized extraction of phenolics from Carob kibbles by Huma et al. [63] who reported 70.11 mg GAE/g dry matter, 68.78 mg GAE/g dry matter, and 69.87 mg GAE/g dry matter using microwave, ultrasound, and conventional solvent, respectively, under the same optimal conditions [62,63].Moreover, Quiles-Carrillo et al. [64] obtained an optimum TPC value of 33.60 mg GAE/g dw by optimized microwave extraction. On the other hand, our results gave higher amounts of TPC compared to the optimum value of TPC of 14.24 mg GAE/g dry Carob matter reported recently by Christou et al. [65].

### 2.3. Effect of Process Variables on Antioxidant Activities (DPPH and TAC)

Table 1 shows the experimental data for antioxidant activities (TAC and DPPH) of Carob pod extracts.

**Figure 4:**
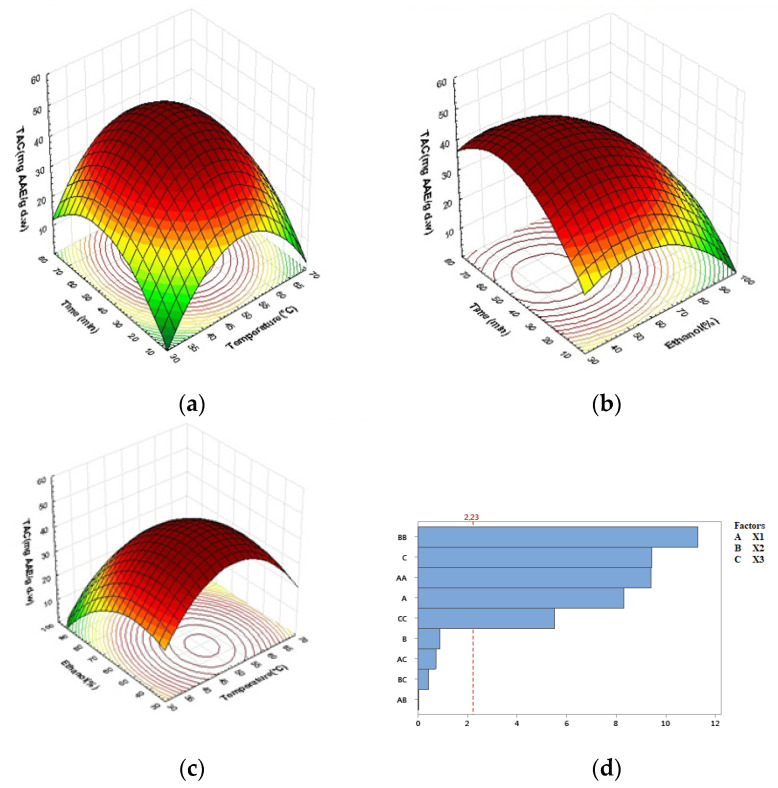
Response surface plots indicating combined effects of UAE variables on TAC: (**a**) time and temperature, (**b**) time and ethanol concentration, (**c**) ethanol concentration and temperature, (**d**) Pareto chart (α = 0.05).

A satisfactory relationship between predicted and experimental data was demonstrated by *R*^2^ values of 97.37% and 96.37% of TAC and DPPH responses, respectively, as shown in Table 3. According to equation models, TAC is significantly affected by quadratic effects of all parameters (*p* < 0.0001), with extraction temperature effects coming in first (Pareto chart in Figure 4d), followed by linear effects of ethanol concentration, quadratic effects of extraction time, linear effects of extraction time, and finally quadratic effects of ethanol concentration.

(2) $$\begin{array}{c} \mathrm{TAC}=-165.4+1.345X_{1}+5.960X_{2}+1.328X_{3}-0.01261X_{1}{}^{2}-0.06058X_{2}{}^{2}-0.01318X_{3}{}^{2}+0.00014X_{1}X_{2}\\ \;\;\;\;\;\;\;\;\;\;\;\;\;\;\;\;\;\;\;\;\;\;\;\;\;\;\;\;\;-0.00175X_{1}X_{3}+0.00218X_{2}X_{3} \end{array}$$

On the other hand, the linear influence of extraction temperature and the interaction terms of all variables were insignificant (*p* > 0.05).

The effect of extraction time and temperature on TAC measured at constant time intervals appeared as saddled shapes on Figure 4a, and it is noticeable that the highest total antioxidant capacity (TAC) was found between 45 and 60 °C within the interval of time between 35 and 50 min.

Figure 4b depicts the effect of ethanol concentration and extraction time. The ethanol concentration (between 30% and 65%) appears to be a more important parameter than the extraction time. The effect of extraction temperature and ethanol concentration is illustrated in Figure 4c; higher temperatures (between 50 and 60 °C) combined with favored ethanol concentrations ranging from 30 to 65 % resulted in higher total antioxidant capacity (TAC). On the other hand, higher ethanol concentrations of 80%, combined with shorter extraction times of 20 min and at a temperature of 40 °C, resulted in the lowest activity (TAC = 22.00 mg AAE/g dw).

For the DPPH method, the ANOVA analysis in Table 3, in addition to the equation model of this response, revealed significant negative effects of all linear (*p* < 0.0001) and major quadratic terms (*p* < 0.01) for the investigated variables (except for extraction temperature). However, interactive terms in this model had a significant (*p* < 0.05) positive effect on DPPH activity.

(3) $$\begin{array}{c} \mathrm{DPPH}=105.2-0.1671X_{1}-0.282X_{2}-0.305X_{3}-0.00954X_{1}{}^{2}-0.00027X_{2}{}^{2}-0.00760X_{3}{}^{2}\\ \;\;\;\;\;\;\;\;\;\;\;\;\;\;\;\;\;\;\;\;\;\;\;\;\;\;\;\;\;+0.00774X_{1}X_{2}+0.01160X_{1}X_{3}+0.00619X_{2}X_{3} \end{array}$$

**Figure 5:**
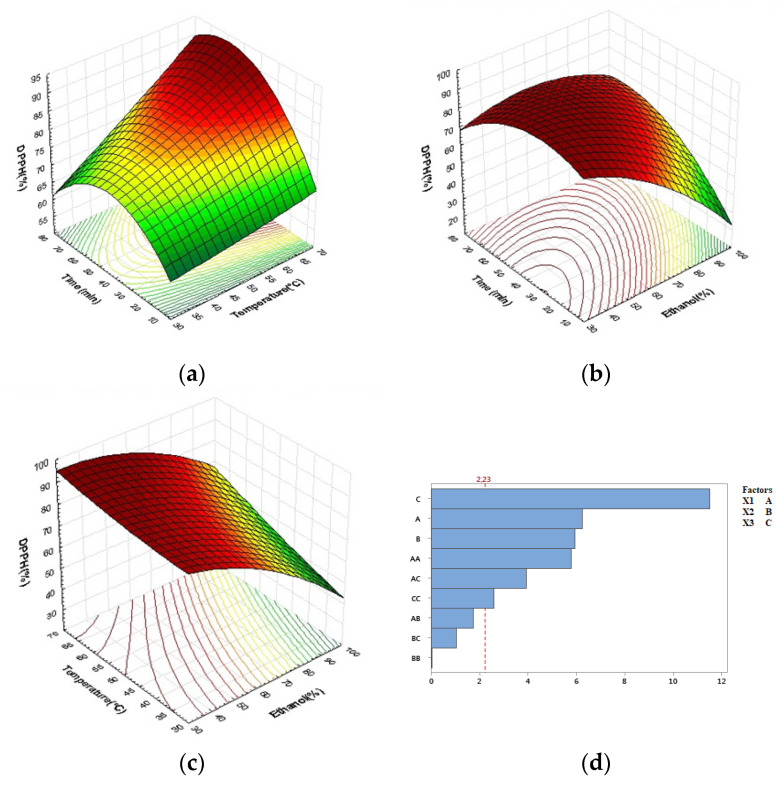
Response surface plots indicating combined effects of UAE variables on DPPH: (**a**) time and temperature, (**b**) time and ethanol concentration, (**c**) temperature and ethanol concentration, (**d**) Pareto chart (α = 0.05).

According to the analysis using the methodology of response surfaces shown in Figure 5, the DPPH activity was strongly affected by ethanol concentration. The results were better (>80%) when a higher temperature was combined with a longer extraction time (less than 70 min), and the ethanol concentration was between 30% and 65%. Higher concentrations of ethanol (>65%) resulted in a decrease in free radical scavenger capacity as measured by the DPPH assay (<70%).

Clearly, increasing the extraction time and temperature at low ethanol concentrations maximizes DPPH activity. Notably, the best antioxidant activities of carob powder extracts, either as DPPH scavenging (90.50%) or total antioxidant capacity TAC (49.50 mg AAE/g dw), were obtained under the same conditions: 50% ethanol concentration and 60 min extraction time, at temperature of 60 °C. This was also observed for the lowest activities, indicating a similar trend for both tested assays.

Furthermore, the findings show that higher antioxidant activity did not correlate with a higher total phenolic content of Carob powder extracts. There is no doubt that Carob extracts have a large spectrum of biological activities, of which the antioxidant capacity is the most studied: these potentials could be attributed to their polyphenolic profile, and our results are in close accordance with data obtained previously [39,40,41,42,43,44,45,46,47,66].

### 2.4. Effect of Process Variables on Photoprotective Activity (SPF)

The photoprotective activity of carob powder extracts was measured in terms of SPF. Based on the equation model and the ANOVA analysis (Table 4) of this response, SPF values were significantly affected by all linear parameters terms (*p* < 0.0001).

(4) $$\begin{array}{c} \mathrm{SPF}=-33.60+0.1795X_{1}+1.437X_{2}+0.284X_{3}-0.000051X_{1}{}^{2}-0.00607X_{2}{}^{2}+0.000737X_{3}{}^{2}\\ \;\;\;\;\;\;\;\;\;\;\;\;\;\;\;\;\;\;\;\;\;+0.00023X_{1}X_{2}-0.001996X_{1}X_{3}-0.01021X_{2}X_{3} \end{array}$$

Furthermore, the model shows a significant influence of quadratic parameters (*p* < 0.05) as well as interactive terms (*p* < 0.0001). The *R*^2^ correlation coefficient of 97.95% indicated that the model fit the experimental results well.

**Figure 6:**
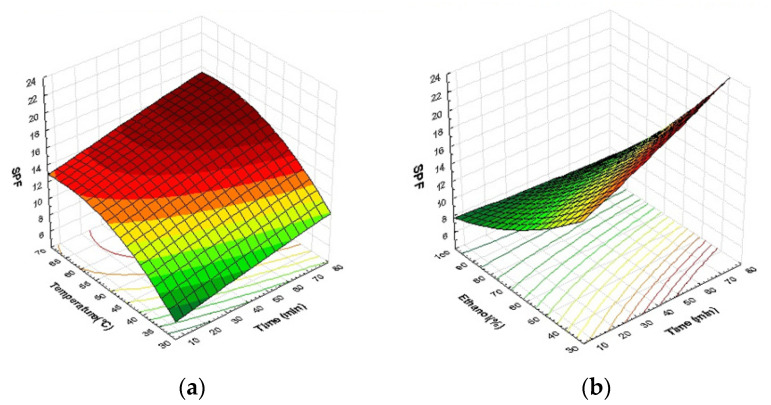

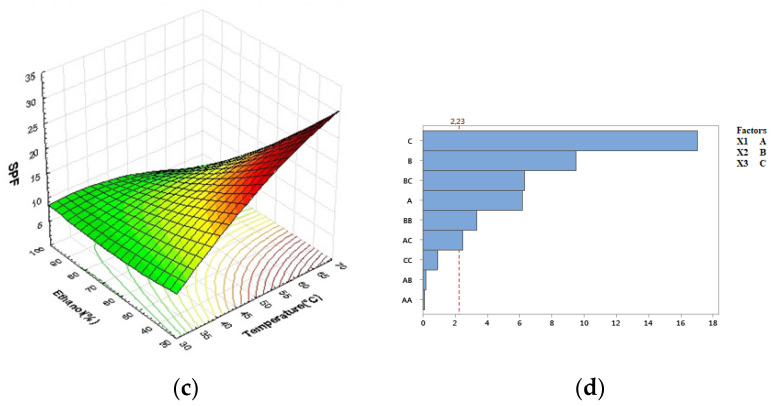
Response surface plots indicating combined effects of UAE variables on SPF: (**a**) time and temperature, (**b**) time and ethanol concentration, (**c**) ethanol concentration and temperature, (**d**) Pareto chart (α = 0.05).

These findings were summarized in a Pareto chart in Figure 6d. The concentration of ethanol had a significant effect on photoprotective activity as measured by SPF. This phenomenon has previously been observed for TPC and DPPH activity. The Pareto chart also shows that SPF measurement was strongly affected by linear terms of extraction time and temperature, as well as quadratic effects of extraction time. The interaction between extraction time and ethanol concentration, along with the quadratic term of ethanol concentration, were ranked last in the classification.

The representation of the SPF response surface supports the preceding conclusions. As shown in Figure 6, higher temperatures (>45 °C) and longer extraction times (>50 min) resulted in a significant photoprotective activity in terms of SPF (SPF > 15). Furthermore, Figure 6b,c show that the main extraction variable was ethanol concentration, and that the best SPF values were obtained at ethanol concentrations ranging from 30% to 50%. At this level, it is worth noting that ethanol with a maximum concentration of 50% could be used in cosmetics [67].

All of the SPF measurement data reported in our experiment design suggests that Carob byproduct extracts have promising sunscreen effects (SPF 8.62 to 22.37). Several studies have shown that medicinal plants, food and their byproducts, and natural polyphenolic antioxidants have a high capacity to protect living organisms from the alterations caused by ROS overproduction. Polyphenolic extracts appear to be particularly promising as cosmetic sunscreens at the moment. Overall, the cosmetic importance of phenolic compounds is based primarily on antioxidant activity. The use of antioxidants in cosmetics reduces oxidative damage, making it a viable option in the treatment and prevention of premature aging. Because of the presence of chromophores in their structure, polyphenols can absorb a wide range of UV radiation, including UVB and UVA. As a result, they prevent solar radiation from penetrating the skin. This feature improves the product’s sun protection and mitigates the negative effects of oxidative stress after sun exposure [13,20,21,22].

According to our experimental design results, the highest value of SPF (SPF 22.37) was obtained under the same conditions that produced the highest level of TPC: 50% ethanol concentration, 60 min of extraction time at temperature of 60 °C. Many consolidated studies in the literature advocate the use of plant, vegetable, fruit and food byproduct extracts with antioxidant activity in photoprotection. Sharma et al. [17] tested five vegetable extracts and five fruit extracts for photoprotective activity by measuring SPF; eggplant and orange extracts showed very high SPF values (SPF 14.37 and SPF 37.40, respectively). Silva et al. [68] also reported the in vitro photoprotective activity of *Spondias purpurea* L. peel extract (SPF 43.78 in dilution of 50 mg/mL). Caballero-Gallardo et al. [69] recently identified the promising photoprotective activities of *Cymbopogon flexuosus* and *Tagetes lucida* with SPF values of 13.40 and 14.70, respectively. It was found that the photoprotective potentials of these natural resources were owing to their higher total phenolic content and antioxidant properties [17,68,69].

Altogether, our experimental design optimization of UAE of Carob byproduct extracts using response surface methodology reveals that the effects of tested parameters (extraction time, extraction temperature, and ethanol concentration) have a similar trend for total phenolics (TPC), antioxidant activities (TAC, DPPH), and photoprotective properties by measuring SPF.

### 2.5. Optimization by RSM

Table 5 shows the optimal UAE conditions for maximum TPC, TAC, DPPH, and SPF responses. The optimal conditions and predicted values were determined using a desirability function with a range of 0.95 to 1, where 1 represents the most desirable response (see Supplementary Material). The experimental confirmation was carried out three times under the optimized conditions obtained from RSM. Table 5 recaps the obtained results.

When the observed experimental and predicted responses were compared, it was discovered that all experimental values were very close to those predicted by the model, ranking within the predicted model’s 95% confidence interval. As a result, it appears reasonable to believe that the model could be successfully applied to the extraction of antioxidant and photoprotective polyphenols from Carob powder extracts. To further validate the results of the photoprotective potential of the optimized final extract (22.127 ± 0.43 of SPF value), its UVA/UVB absorbance ratio was calculated. Optimized Carob byproduct extracts demonstrated a good UVA screen (0.47 UVA/UVB ratio, 2 stars) by applying the Boots Star Rating system, indicating that our prepared extract is a potential photoprotective agent for use as an alternative active ingredient in sunscreen commercial formulations for cosmetics.

## 3. Materials and Methods

### 3.1. Raw Material and Chemicals

**Figure 7:**
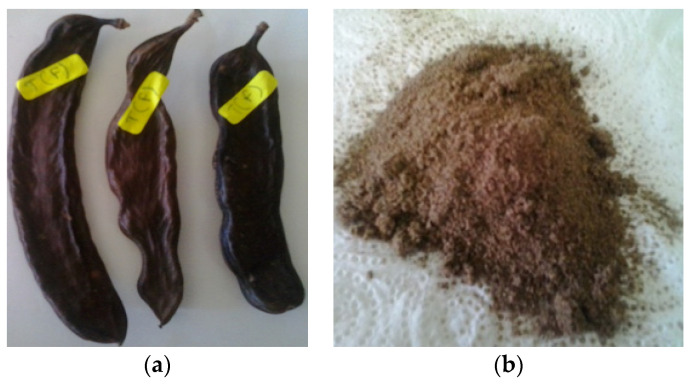
(**a**) Carob pods; (**b**) final Carob byproducts powder.

Carob pods (pulp and seeds) were obtained from the fruit of a carob tree (*Ceratonia siliqua* L.) located in the region of Texenna (Jijel, North east of Algeria) (Figure 7a). Pods (pulp and seeds) were cut in small pieces and ground using an electric grinder (Retsch, GRINDOMIX GM 200). The process of grinding was repeated several times in order to obtain a fine Carob powder (Figure 7b).

Ascorbic and gallic acids were purchased from Sigma-Aldrich (St. Louis, MO, USA) and used as standards for calibration curves. Other chemicals: 1,1-diphenyl-2-picrylhydrazyl (DPPH), ammonium molybdate tetrahydrate, and the Folin–Ciocalteu reagent were purchased from Sigma-Aldrich (Steinheim, Germany). Other solvents and reagents used were of analytical grade.

### 3.2. Preliminary Assays

The preliminary experiments were carried out in order to select the efficient extraction process of phytoantioxidants from Carob byproducts, through a comparison between the results of maceration (at 25 °C) and heating-assisted extraction (at 50 °C) as conventional methods, and ultrasound assisted extraction (at 25 °C and 50 °C) as a green method. This methodology also allowed us to determine whether thermal processing and ultrasonic influences affect Carob byproducts extraction efficiency. The extraction efficiency was expressed via four responses: total polyphenol contents (TPC), total antioxidant capacity (TAC), DPPH scavenging activity, and photoprotective activity by measuring SPF.

As our work aimed to valorize Carob byproducts in the cosmetic industry, ethanol was chosen as an extraction solvent. Ethanol is non-toxic and can be considered an environmentally benign solvent.

To begin, we used a maceration method for 60 min at 25 °C to select a suitable solvent-to-solid ratio and solvent concentration. To that end, samples (1 g) were macerated with 50% ethanol by considering three ratios (30/1, 20/1, and 10/1; *v*/*m*) for 60 min at 25 °C. After this the optimal ethanol concentration for best solvent-to-solid ratio was investigated: 50% ethanol, 70% ethanol, and 100% ethanol. The maceration lasted 60 min at 25 °C.

After defining the best solvent-to-solid ratio and its concentration, heating-assisted extraction (at 50 °C) and UAE (at 25 °C and 50 °C) for 60 min were performed to complete our methodology.

On the basis of the results of those procedures, the central composite design rotatable (CCDR) was developed with the aim of optimizing the most efficient extraction process.

### 3.3. Ultrasound-Assisted Extraction Process Optimization

Independent parameters were selected on the basis of the results of our preliminary assays and of previous research regarding the extraction of phenolic compounds from Carob extracts, which are associated with the plant material antioxidant activity [60,62,63,70,71]. The afore-mentioned researchers suggested that the solvent concentration, the extraction time, and the extraction temperature were the variables with the most influence on the extraction process. Moreover, using a lower solvent-to-solid ratio, Carob extracts with higher yields of antioxidants are achieved.

Thus, in all experimental runs, 1 g of powdered Carob was mixed with 10 mL of the extraction solvent in screw-cap tubes and sonicated for various times and temperatures as required by the experiment (Table 1) using an ultrasonic cleaning bath (ultrasons-H, 50/60 Hz, 720 W, Ctra. Nll Km: 585.1 Abrera, Barcelona—Spain). Following extraction and cooling, samples were filtered through Whatman No.1 paper and subjected to various analyses.

### 3.4. Experimental Design and Statistical Analysis

A central composite design rotatable CCDR was used on three independent variables across two levels, with six axial points and six replicates of the central point, for a total of 20 extractions [72]. Table 6 shows the coding values, levels, and real values. Table 1 shows the experimental parameters and Y responses for each test, as well as the corresponding averages.

Using the obtained results, the regression coefficients can be calculated and the equation that best fits each test performed in this study can be constructed using the following equation:

(5) $$Y={\;\alpha }_{0\;}+\overset{n}{\underset{i=1}{\sum }}\alpha_{i}X_{i}+\overset{n}{\underset{i=1}{\sum }}\alpha_{\mathrm{ii}}X_{i}^{2}+\overset{n}{\underset{i=1}{\sum }}\overset{n}{\underset{j=1}{\sum }}\alpha_{\mathrm{ij}\;}X_{i}X_{J}$$

where Y represents the predicted response value (TPC, TAC, DPPH, SPF); X_1_, X_2_, X_3_ are independent factors (extraction time, solvent concentration, and extraction temperature); α_0_ signifies the theoretical mean value of the response when all factors are at level 0; α_i_ denotes linear regression coefficients; α_ii_ denotes quadratic regression coefficients; and α_ij_ denotes interaction regression coefficients.

### 3.5. Determination of Total Phenolic Content (TPC)

The TPC of samples was estimated using Folin–Ciocalteu reagent [73]. Briefly, 0.30 mL of diluted extract was mixed with 1.20 mL of Folin-Ciocalteu reagent (diluted 1:10). After 5 min, 1.50 mL of a 7.50 % Na_2_CO_3_ solution was added to the mixture. The tubes were incubated at room temperature in the dark for 2 h. A UV/visible spectrophotometer was used to measure the absorbance at 765 nm. The TPC was calculated by extrapolating the calibration curve, which was created by preparing a gallic acid solution (0–200 μg/mL). The test was carried out in triplicate and the results were presented as mg of gallic acid equivalents per dry weight of sample (GAE mg/g dw).

### 3.6. Total Antioxidant Capacity (TAC)

The TAC of extracts was calculated using the phosphomolybdate method [74]. First, a reagent solution was prepared (0.60 M sulfuric acid, 28 mM sodium phosphate, and 4 mM ammonium molybdate). A 0.30 mL aliquot of the extract solution was mixed with 3 mL of the reagent solution. The mixture was incubated at 95 °C for 90 min. After cooling, the absorbance was measured at 695 nm against a blank. The antioxidant capacity of the samples was measured in milligrams of ascorbic acid equivalent (mg AAE/g dw). All assays were carried out in triplicate.

### 3.7. Scavenging Activity of DPPH Radical

The free radical scavenging activity of our extract was determined using a modified method from the literature [75,76,77,78,79,80,81]. Each 0.50 mL aliquot of the sample extract was mixed with 3.50 mL of DPPH ethanolic solution. After 30 min in the dark, the absorbance at 517 nm was measured against ethanol.

The percentage of inhibition (I%) was calculated as follows:

(6) $$I\%=\left(\frac{\mathrm{Abs}.\mathrm{of}\;\mathrm{control}-\mathrm{Abs}.\mathrm{of}\;\mathrm{sample}}{\mathrm{Abs}.\mathrm{of}\;\mathrm{control}}\right)\times 100$$

The control was prepared without any sample under the same conditions. All dilutions were performed in triplicates.

### 3.8. Photoprotective Activity (Measurement of SPF)

Mansur’s method was used to calculate the in vitro sun protection factor (SPF) [82]. Mansur’s method is a straightforward spectrophotometric analysis that is easily reproducible. SPF was used to assess the photoprotective activity of diluted extract solutions. The absorption spectra of the sample solution were obtained every 5 nm using a standard 1 cm quartz cell and ethanol as the blank (UV/Visible spectrophotometer, Thermo Electron Corporation Evolution 100). Triplicates were made, followed by the application of the Mansur equation:

(7) $$\mathrm{SPF}=\mathrm{CF}\cdot \overset{320}{\underset{290}{\sum }}\mathrm{EE}\left(\lambda \right)\cdot I\left(\lambda \right)\cdot \mathrm{Abs}\left(\lambda \right)$$

where the correction factor (CF) is 10, EE (λ) is the erythemogenic effect of radiation on wavelength λ, I (λ) is the intensity of solar light with wavelength λ, and Abs is the sample spectrophotometric absorbance value at wavelength λ. The values of EE (λ) × I (λ) are constants determined by [83]. The optimized extract prepared from Carob byproducts was also screened for its UVA/UVB absorbance ratio according to the Boots Star Rating system, another in vitro parameter to evaluate photoprotective activity. According to this ratio, calculated with Equation (8), the star rating system indicates that the UVA/UVB ratio in the range 0.0 to 0.2 is too low for UVA protection (−), 0.2 to 0.4 is a moderate protector (*), 0.4 to 0.6 is a good protector (**), 0.6 to 0.8 is a superior protector (***), and ≥0.8 is a maximum protector (****) [69].

(8) $$\mathrm{UVA}/\mathrm{UVB}\;\mathrm{ratio}=\frac{\int_{320}^{400}A_{\lambda }d\lambda /\int_{320}^{400}d\lambda }{\int_{290}^{320}A_{\lambda }d\lambda /\int_{290}^{320}d\lambda }$$

### 3.9. Statistical Analysis

Multiple regression analysis was performed using Minitab Release 19 (Minitab Inc., State College, PA, USA). The models were used to determine response surfaces in Statistica 10 (Stat soft, Paris, France). One way analysis of variance (ANOVA) was applied to compare the effect of selected variables on the responses. A coefficient of determination (*R*^2^) was computed and the adequacy of models was tested by separating the residual sum of squares into pure error and lack-of-fit. Optimization was also performed with Minitab Release 19 (Minitab Inc., State College, PA, USA).

## 4. Conclusions

Rising concern over the safety of cosmetics, in particular sunscreens, has led to an increased interest in the exploration of effective and economical natural photoprotectors and antioxidants. In this context, byproducts of food processing industries assume significance because of their accessibility, nontoxicity, and availability in large quantities. Carob byproducts could be a good commercial source of antioxidants with photoprotective activity. As a preliminary step in this study, the efficacy of ultrasonic-assisted extraction (UAE) of phytoantioxidants from Carob byproducts versus conventional methods was demonstrated. The next step was the optimization of the extraction conditions: ethanol concentration (%), time extraction (min), and temperature extraction (°C) by using response surface methodology (RSM); this allowed the best conditions to be found to maximize the targeted responses: TPC, TAC, DPPH, and photoprotective activity by measuring SPF. Statistical and graphical data provided indicate that ethanol concentration was the most influential parameter in the extraction of all responses apart from total antioxidant capacity (TAC). The optimal UAE parameters for maximum responses within this experimental design process were an ethanol concentration of 38.90%, an extraction time of 50.52 min, and an extraction temperature of 53.90 °C. Our model was validated according to the findings of a desirability function. Interestingly, an optimized extract made from Carob byproducts demonstrated a good UVA screen with a ratio of 0.47 UVA/UVB . Combining Carob byproducts as a source of sustainable and bioactive products with optimized UAE is a promising contribution to the cosmetic industry that will help to strengthen the concept of both sustainability and valorization of these agri-food waste materials as active ingredients for innovative phytocosmetic formulations.

## Figures and Tables

**Table 1:** Central composite design rotatable (CCDR) of three variables at three levels, and the resultant responses: TPC, TAC, DPPH, and SPF.

Entry	UAE Independent Variables			Investigated Responses			
	X_1_ (min)	X_2_ (°C)	X_3_ (%)	TPC (mg GAE/g dw)	TAC (mg AAE/g dw)	DPPH (%)	SPF
1	40	50	65	15.33	48.19	85.46	14.08
2	20	60	50	14.04	31.76	86.99	18.23
3	20	60	80	6.21	26.76	63.78	10.45
4	40	50	65	15.21	46.92	81.13	13.99
5	60	60	80	10.33	33.90	82.97	10.74
6	40	50	65	15.04	48.57	81.56	13.63
7	60	60	50	21.92	46.92	89.47	22.37
8	40	50	65	15.17	48.97	81.34	13.75
9	20	40	80	7.33	22.00	56.35	8.62
10	40	50	65	15.00	48.74	81.67	13.47
11	60	40	50	13.62	43.35	79.56	14.23
12	40	50	65	15.29	47.86	81.88	14.06
13	73.60	50	65	12.87	39.22	80.76	15.98
14	40	66.80	65	12.25	31.52	87.88	14.28
15	60	40	80	11.67	34.94	66.56	10.18
16	6,4	50	65	6.88	28.43	64.84	11.73
17	40	33.20	65	10.30	30.41	79.10	10.12
18	40	50	39.80	21.58	49.30	90.50	19.31
19	20	40	50	10.29	34.22	80.49	11.73
20	40	50	90.20	11.12	30.09	66.98	9.45

GAE—gallic acid equivalents, AAE—ascorbic acid equivalents, dw—dry weight, X_1_—extraction time, X_2_—extraction temperature, and X_3_—ethanol concentration.

**Table 2:** ANOVA for evaluation of the total phenolic content (TPC).

Source of Variation		DF	Sum of Squares	F-Value	*p*-Value
Model		9	320.074	56.90	0.000
Linear		3	205.605	109.65	0.000
X_1_		1	64.791	103.66	0.000
X_2_		1	12.132	19.41	0.001
X_3_		1	128.682	205.88	0.000
Quadratic		3	84.862	45.26	0.000
X_1_ × X_1_		1	55.362	88.57	0.000
X_2_ × X_2_		1	30.919	49.47	0.000
X_3_ × X_3_		1	1.574	2.52	0.144
Interaction		3	29.606	15.79	0.000
X_1_ × X_2_		1	2.344	3.75	0.082
X_1_ × X_3_		1	0.945	1.51	0.247
X_2_ × X_3_		1	26.318	42.11	0.000
Error		10	6.250		
Lack of fit		5	6.163	70.57	0.000
Pure error		5	0.087		
Total		19	326.324		
*R* ^2^	98.08%	*R* ^2^_(adj)_		96.36%	

**Table 3:** ANOVA for evaluation of the total antioxidant capacity (TAC) and DPPH method.

	TAC (mg AAE/g dw)				DPPH (%)			
Source of Variation	DF	Sum of Squares	F-Value	*p*-Value	DF	Sum of Squares	F-Value	*p*-Value
Model	9	1532.26	41.17	0.000	9	1655.91	29.47	0.000
Linear	3	658.18	53.06	0.000	3	1294.78	69.13	0.000
X_1_	1	286.26	69.23	0.000	1	243.96	39.08	0.000
X_2_	1	3.28	0.79	0.394	1	221.70	35.51	0.000
X_3_	1	368.64	89.15	0.000	1	829.12	132.81	0.000
Quadratic	3	871.00	70.22	0.000	3	238.09	12.71	0.001
X_1_ × X_1_	1	365.53	88.40	0.000	1	209.04	33.49	0.000
X_2_ × X_2_	1	527.13	127.49	0.000	1	0.01	0.00	0.968
X_3_ × X_3_	1	126.25	30.53	0.000	1	42.01	6.73	0.027
Interaction	3	3.07	0.25	0.861	3	123.04	6.57	0.010
X_1_ × X_2_	1	0.01	0.00	0.969	1	19.19	3.07	0.110
X_1_ × X_3_	1	2.22	0.54	0.481	1	96.95	15.53	0.003
X_2_ × X_3_	1	0.85	0.21	0.660	1	6.90	1.11	0.318
Error	10	41.35			10	62.43		
Lack of fit	5	38.57	13.90	0.006	5	49.13	3.69	0.089
Pure error	5	2.78			5	13.30		
Total	19	1573.61			19	1718.34		
	*R* ^2^	97.37%	*R* ^2^_(adj)_	95.01%	*R* ^2^	96.37%	*R* ^2^_(adj)_	93.10%

**Table 4:** ANOVA for evaluation of measurement of SPF.

Source of Variation		DF	Sum of Squares	F-Value	*p*-Value
Model		9	224.216	53.08	0.000
Linear		3	196.545	139.59	0.000
X_1_		1	17.904	38.15	0.000
X_2_		1	42.280	90.09	0.000
X_3_		1	136.360	290.55	0.000
Quadratic		3	6.029	4.28	0.035
X_1_ × X_1_		1	0.006	0.01	0.913
X_2_ × X_2_		1	5.286	11.26	0.007
X_3_ × X_3_		1	0.395	0.84	0.381
Interaction		3	21.643	15.37	0.000
X_1_ × X_2_		1	0.017	0.04	0.852
X_1_ × X_3_		1	2.868	6.11	0.033
X_2_ × X_3_		1	18.758	39.97	0.000
Error		10	4.693		
Lack of fit		5	4.376	13.81	0.006
Pure error		5	0.317		
Total		19	228.910		
*R* ^2^	97.95%	*R* ^2^_(adj)_		96.10%	

**Table 5:** Estimated optimal conditions: predicted and experimental values of investigated responses.

Optimum UAE Parameters							
Time (min)		Temperature (°C)				Ethanol (%)	
50.52		53.90				38.90	
**Response variables**							
**TPC (mg GAE/g dw)**		**TAC (mg AAE/g dw)**		**DPPH (%)**		**SPF**	
Predicted	Experimental *	Predicted	Experimental	Predicted	Experimental	Predicted	Experimental
23.632	23.375 ± 0.83	49.10	48.89 ± 0.24	89.89	89.27 ± 0.95	22.348	22.127 ± 0.43

* Values are means ±SD of three independent replicates.

**Table 6:** Levels of coded and real independent variables used in the experimental design (CCDR).

Independent Variable	Code	Factors Levels				
		−α ^1^	−1	0	1	+α
Extraction time (min)	X_1_	6.40	20	40	60	73.60
Temperature (°C)	X_2_	33.20	40	50	60	66.80
Ethanol concentration (%)	X_3_	39.80	50	65	80	90.20

^1^ α (axial distance) = 1.68.

## Data Availability

Not applicable.

## Supplementary Materials

The following supporting information can be downloaded at: https://www.mdpi.com/article/10.3390/molecules27248802/s1, Figure S1: Determination of optimal conditions and predicted responses (TPC, TAC, DPPH, and SPF) by desirability function; Table S1: Optimization of the parameters by desirability function; Table S2: Solution of desirability function; Table S3: Optimal conditions and predicted responses by desirability function; Table S4: Relationship between the erythemogenic effect and the intensity of the radiation at each wavelength.
