# Supplementary material for: Process Optimization of Phytoantioxidant and Photoprotective Compounds from Carob Pods (Ceratonia siliqua L.) Using Ultrasonic Assisted Extraction Method
Source: Molecules. 2022 Dec 12;27(24):8802. doi: 10.3390/molecules27248802 (PMC9783530; doi:10.3390/molecules27248802)
Supplement: Supplementary file 1 [file molecules-27-08802-s001.zip › molecules-2048558-supplementary.pdf]

Supplementary Materials

Article

Process optimization of phytoantioxidant and photoprotective compounds from Carob pods (*Ceratonia siliqua* L.) using ultrasonic assisted extraction method

Radia Ayad [1, 2], Rima Ayad [3], Hayat Bourekoua[4], Mostefa Lefahal[1], El Hani Makhloufi[1], Salah Akkal[1], Kamel Medjroubi [1] and Gema Nieto[5,*]

[1] Valorization of Natural Resources, Bioactive Molecules and Biological Analysis Unit, Department of
Chemistry, Frères Mentouri Constantine 1 University, 25000 Constantine, Algeria; radia.ayad@univ-jijel.dz (R.A.); mlefahal@gmail.com (M.L.); makhloufi_el_hani@yahoo.fr (E.H.M.); salah4dz@yahoo.fr (S.A.); medjroubi@yahoo.fr (K.M.)
[2] Laboratory of Phytochemistry and Pharmacology, Department of Chemistry, Faculty of Exact Sciences and Informatics, University Mohammed Seddik Benyahia of Jijel, 18000 Jijel, Algeria
[3] Laboratory of Biotechnology, Environment and Health, Department of Applied Microbiology and Food Science, Faculty of Nature and Life Sciences, University Mohammed Seddik Benyahia of Jijel,
18000 Jijel, Algerie; rima.ayad@univ-jijel.dz
[4] Laboratoire de Nutrition et Technologie Alimentaire (LNTA), Institut de la Nutrition, de l'Alimentation et des Technologies Agro-Alimentaires (INATAA), Frères Mentouri-Constantine 1 University, 25000 Constantine, Algeria;bourekoua.h@hotmail.fr
[5] Department of Food Technology, Food Science and Nutrition, Faculty of Veterinary Sciences, Regional Campus of International Excellence "Campus Mare Nostrum", University of Murcia, 30071 Murcia, Spain
* Correspondence: gnieto@um.es; Tel.: +34-868-889-624

Contents

I. Supplementary Table S1. Optimization of the parameters by desirability function (optimisation des paramètres)

II. Supplementary Table S2. Solution of desirability function

III. Supplementary Table S3. Optimal conditions and predicted responses by desirability function (Multiple response predictions)

IV. Supplementary Table S4. Relationship between the erythemogenic effect, and the intensity of the radiation at each wavelength

V. Supplementary Figure S1. Determination of optimal conditions and predicted responses (TPC, TAC, DPPH, and SPF) by desirability function

I. Supplementary Table S1. Optimization of the parameters by desirability function (optimisation des paramètres)

Response	Objective	Lower	Target	higher	Weight	Importance
SPF	Maximum	8.62	22.37		1	1
DPPH	Maximum	56.35	90.50		1	1
TAC	Maximum	22.00	49.30		1	1
TPC	Maximum	6.21	21.92		1	1

II. Supplementary Table S2. Solution of desirability function

Solution	X1	X2	X3	SPF Adjusted value	DPPH Adjusted value	TAC Adjusted value	TPC Adjusted value	Desirability composite
1	50.5212	53.9030	39.8	22.3477	89.8908	49.1048	23.6322	0.993322

III. Supplementary Table S3. Optimal conditions and predicted responses by desirability function (Multiple response predictions)

Variable	Configuration
X1	50.5212
X2	53.903
X3	39.8

Response	Adjusted value	ErT ajust	IC at 95 %	IP at 95 %
SPF	22.348	0.601	(21.008; 23.687)	(20.317; 24.379)
DPPH	89.89	2.19	(85.00; 94.78)	(82.48; 97.30)
TAC	49.10	1.78	(45.13; 53.08)	(43.08; 55.13)
TPC	23.632	0.694	(22.086; 25.178)	(21.288; 25.976)

IV. Supplementary Table S4. Relationship between the erythemogenic effect, and the intensity of the radiation at each wavelength

λ (nm)	290	295	300	305	310	315	320	Total
EE (λ) × I (λ)	0.0150	0.0817	0.2874	0.3278	0.1864	0.0839	0.0180	1.0000

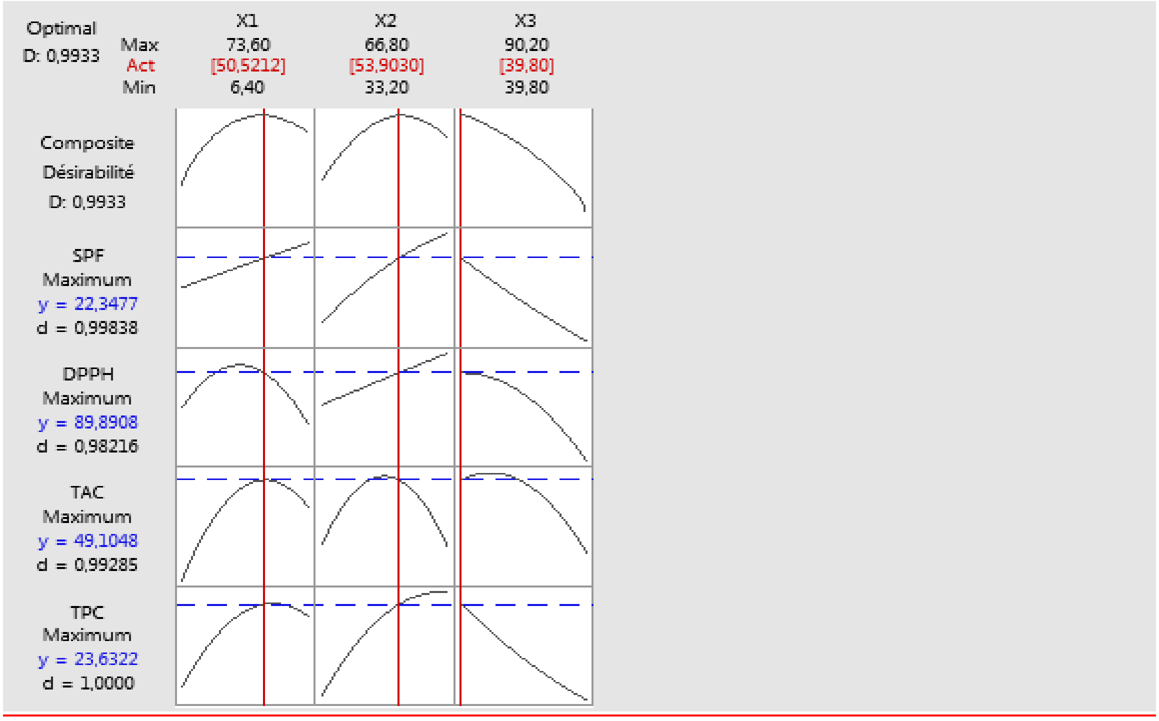

V. Supplementary Figure S1. Determination of optimal conditions and predicted responses (TPC, TAC, DPPH, and SPF) by desirability function
